# In-vivo inhibition of neutral endopeptidase 1 results in higher absorbed tumor doses of [^177^Lu]Lu-PP-F11N in humans: the lumed phase 0b study

**DOI:** 10.1186/s13550-024-01101-w

**Published:** 2024-04-06

**Authors:** Christof Rottenburger, Michael Hentschel, Markus Fürstner, Lisa McDougall, Danijela Kottoros, Felix Kaul, Rosalba Mansi, Melpomeni Fani, A. Hans Vija, Roger Schibli, Susanne Geistlich, Martin Behe, Emanuel R. Christ, Damian Wild

**Affiliations:** 1 Division of Nuclear Medicine, University Hospital Basel, Petersgraben 4, 4031 Basel, Switzerland; 2 Division of Medical Radiation Physics, Department of Radiation Oncology, Bern University Hospital, Bern, Switzerland; 3 Center for Neuroendocrine and Endocrine Tumors, University Hospital Basel, Basel, Switzerland; 4 Division of Radiopharmaceutical Chemistry, University Hospital Basel, Basel, Switzerland; 5 Molecular Imaging, Siemens Medical Solutions USA, Inc., Hoffman Estates, IL USA; 6 Department of Chemistry and Applied Biosciences, Institute of Pharmaceutical Sciences, ETH, Zurich, Switzerland; 7 Center for Radiopharmaceutical Sciences, Paul Scherrer Institute, Villigen, Switzerland; 8 Division of Endocrinology, Diabetology and Metabolism, University Hospital Basel, Basel, Switzerland

**Keywords:** Neutral endopeptidase 1, Endopeptidase inhibition, Cholecystokinin-2 receptor targeting, Peptide receptor radionuclide therapy, [^177^Lu]Lu-PP-FF11N

## Abstract

**Background:**

A new generation of radiolabeled minigastrin analogs delivers low radiation doses to kidneys and are considered relatively stable due to less enzymatic degradation. Nevertheless, relatively low tumor radiation doses in patients indicate limited stability in humans. We aimed at evaluating the effect of sacubitril, an inhibitor of the neutral endopeptidase 1, on the stability and absorbed doses to tumors and organs by the cholecystokinin-2 receptor agonist [^177^Lu]Lu-PP-F11N in patients. In this prospective phase 0 study eight consecutive patients with advanced medullary thyroid carcinoma and a current somatostatin receptor subtype 2 PET/CT scan were included. Patients received two short infusions of ~ 1 GBq [^177^Lu]Lu-PP-F11N in an interval of ~ 4 weeks with and without Entresto^®^ pretreatment in an open-label, randomized cross-over order. Entresto^®^ was given at a single oral dose, containing 48.6 mg sacubitril. Adverse events were graded and quantitative SPECT/CT and blood sampling were performed. Absorbed doses to tumors and relevant organs were calculated.

**Results:**

Pretreatment with Entresto^®^ showed no additional toxicity and increased the stability of [^177^Lu]Lu-PP-FF11N in blood significantly (*p* < 0.001). Median tumor-absorbed doses were 2.6-fold higher after Entresto^®^ pretreatment (0.74 vs. 0.28 Gy/GBq, *P* = 0.03). At the same time, an increase of absorbed doses to stomach, kidneys and bone marrow was observed, resulting in a tumor-to-organ absorbed dose ratio not significantly different with and without Entresto^®^.

**Conclusions:**

Premedication with Entresto^®^ results in a relevant stabilization of [^177^Lu]Lu-PP-FF11N and consecutively increases radiation doses in tumors and organs.

*Trial registration* clinicaltrails.gov, NCT03647657. Registered 20 August 2018.

## Background

About 90% of medullary thyroid carcinomas (MTCs) express the transmembrane G protein–coupled cholecystokinin 2 receptor (CCK2R) at a high density [1], making CCK2R an interesting target for peptide receptor radionuclide therapy (PRRT). Recently, a new generation of radiolabeled minigastrin derivatives, targeting CCK2R, with improved and promising properties was introduced [2]. First clinical trials explore the potential of such compounds for imaging and PRRT of CCK2R expressing tumors, in particular for MTC [3–5]. However, the reported tumor radiation doses were lower than those usually achieved by PRRT using somatostatin receptor (SSTR) targeting ligands in patients with non-MTC neuroendocrine tumors [3]. Beyond factors such as low target expression on the tumor surface, a limited stability of the radiopharmaceutical might be the reason for a low tumor uptake. When using enzymatic unstable peptide receptor targeted radiopharmaceuticals, co-injection of peptidase inhibitors such as phosphoramidon, an inhibitor of neutral endopeptidase 1 neprilysin (NEP), can result in a significant increase in tumor uptake in vivo in animals [6]. However, this effect was not significant in an athymic mouse model for the peptidase inhibitor phosphoramidon and only to a minor extent for thiorphan when co-injected with the moderately stable [^177^Lu]Lu-DOTA-(DGlu)_6_-Ala-Tyr-Gly-Trp-Met-Asp-Phe-NH_2_ ([^177^Lu]Lu-PP-F11N) [7]. The limited value of in-vitro stability testing was previously reported for several minigastrin derivatives, revealing a much more rapid degradation in-vivo than expected from in-vitro studies [8]. This could be explained by the different conditions of exposure to degrading enzymes in-vitro compared to the physiological conditions in the blood circulation. In particular, the abundant and ubiquitous presence of NEP within the human body [9, 10] may explain the overestimation of stability in in-vitro testing. Similarly, limitations in the predictive value of preclinical in-vivo stability testing, e.g. caused by species-specific expression of proteases [11], cannot be excluded and may be difficult to detect, particularly for a moderately stable compound such as [^177^Lu]Lu-PP-F11N. Clinical in-vivo evaluation of a compound would then be the only way to identify limited in-vivo stability or an additional benefit of peptidase inhibition for PRRT.

Sacubitril (AHU377) is a potent inhibitor of the NEP. The combination of sacubitril and the angiotensin-receptor blocker valsartan (Entresto^®^) is effective in reducing the risks of death and of hospitalization for heart failure [12] and is approved for this indication in numerous countries. The highest blood plasma concentration of its active metabolite, LBQ657, is measured around two hours after oral intake [13]. Therefore, it is hypothesized that pretreatment with Entresto^®^ 2 h before injection will increase the stability of [^177^Lu]Lu-PP-F11N and consecutively the tumor-absorbed dose.

The primary endpoint of this study was the determination of the tumor-absorbed dose of [^177^Lu]Lu-PP-F11N with and without Entresto^®^ pretreatment. Furthermore, we determined the adverse effects of this combination and the effect of the oral intake of Entresto^®^ on the in-vivo stability of [^177^Lu]Lu-PP-F11N in patients as well as the absorbed doses to tumor and organs. Finally, CCK2R tumor imaging ([^177^Lu]Lu-PP-F11N SPECT/CT) was compared with somatostatin receptor subtype 2 (SSTR2) PET/CT imaging ([^68^Ga]Ga-DOTATOC or [^68^Ga]Ga-DOTATATE) in all patients.

## Material and methods

### Study design and patients

This is a prospective phase 0, single-center imaging and dosimetry study (ClinicalTrials.gov: NCT03647657) in order to evaluate the effect of Entresto^®^ pretreatment on tumor-absorbed dose of [^177^Lu]Lu-PP-F11N in the same patients using an open-label randomized cross-over design. The study was approved by the ethics committee of Northwest and Central Switzerland and performed in accordance with good clinical practice standards. Patients were recruited between December 2018 and October 2021 in a consecutive order and signed a written informed consent form in accordance with the Declaration of Helsinki. Except for the inclusion and exclusion criteria, there was no further selection of participating patients and the expression status of CCK2R was not evaluated previously.

Inclusion criteria: Advanced MTC with elevated levels of calcitonin (> 100 pg/ml) and/or calcitonin-doubling time < 24 months before or after total thyroidectomy. SSTR2 targeted PET/CT not older than 12 weeks, Age > 18 years and informed consent.

Exclusion Criteria: Medication with kinase inhibitors 3 weeks before the study and during the study. Renal failure (calculated glomerular filtration rate < 60 ml/min per 1.73 m^2^ body surface). Bone marrow failure (thrombocytes < 70,000/μl, leucocytes < 2500/μl, hemoglobin < 8 g/dl). Pregnancy and breast feeding. Known, serious side reaction in the case of a former application of pentagastrin, active, second malignancy or remission after second malignancy < 5 years.

To avoid possible side effects, according to the package insert of Entresto^®^: Patients at an age of more than 64 years and/or with systolic blood pressure < 112 mmHg at the time of screening. Simultaneous medication with angiotensin converting enzyme (ACE)-inhibitors, or withdrawal for less than 36 h prior to the medication with Entresto^®^ or simultaneous medication with AT-II-receptor blockers. Known intolerance to Sacubitril or Valsartan. Known angioedema in the context of a medication with an ACE-inhibitor or an AT-II-receptor blocker.

### Study procedure

Patients were allowed a light breakfast at the day of each infusion. [^177^Lu]Lu-PP-F11N was produced as previously described [3]. Each patient received two short infusions (4 min) of 1.08 ± 0.05 GBq (70.3 ± 7.3 µg) [^177^Lu]Lu-PP-F11N (mean ± SD) in an interval of 28.9 ± 2.5 days with and without Entresto^®^ (Novartis Pharma Schweiz AG, Rotkreuz, Switzerland) premedication in a randomized cross-over order. Entresto^®^ was given at an oral dose of 100 mg, containing 48.6 mg sacubitril and 51.4 mg valsartan 2 h before the start of one of the two infusions. Vital signs were recorded, and a 12-lead electrocardiogram was obtained before and after each injection. Adverse events were recorded and graded according to Common Terminology Criteria for Adverse Events (CTCAE) version 4.03.

### In-vivo stability

Blood samples were collected in polypropylene tubes containing ethylenediaminetetraacetic acid at 5, 15, 30 and 60 min after administration of ^177^Lu-PP-F11N. The tubes were placed on ice. Plasma was separated by centrifugation (4000 g for 10 min) and protein precipitation followed using cold methanol (v/v 1/2). The mixture was stirred to favor precipitation of proteins and then centrifuged at 4000 g for 10 min. The supernatant was collected and filtered through a 0.22 µM filter (Millex^®^-GV, Merck Millipore, Bedford, MA, USA). The clear solution was diluted with H_2_O (1:1) and then analyzed by radio-high performance liquid chromatography (HPLC) to determine the percentage of intact peptide over time. HPLCs were performed on the Agilent 1260 infinity instrument (Agilent, Santa Clara, United States) connected to a GABI radioactivity-HPLC-flow-monitor γ-spectrometer (Elysia-raytest, Straubenhardt, Germany). Radioligands were analyzed using Phenomenex Jupiter Proteo C12 (90 Å, 250 × 4.6 mm) column (Phenomenex, Torrance, USA) using the gradient 5–50% B in 15 min (A = H_2_O [0.1% trifluoroacetic acid], B = ACN [0.1% trifluoroacetic acid]) with a flow rate of 2 mL/min.

### SPECT/CT and SPECT/CT based dosimetry, PET/CT imaging and reading

Planar whole-body scintigraphy and quantitative SPECT/CT acquisitions of neck/thorax/abdomen/pelvis were acquired with a hybrid SPECT/CT system (Siemens Intevo, Siemens Erlangen, Germany) at 1, 4, 24 and 72 h post injection (p.i.).

Calibration of the SPECT/CT camera was performed with a National Institute of Standards and Technology traceable selenium-75 point source (uncertainty in activity 3%) every four weeks. Acquisitions (medium-energy low-penetration collimator) were performed in step and shoot mode using 20 s/view, 64 views/head using a non-circular orbit with a 180° detector configuration in a 128 × 128 matrix. The photo-peak window was set to 208 keV ± 10% (187.2–228.8 keV). Except for the first patient, the quantitative tomographic images (isotropic voxel size 5.08 mm^3^) were reconstructed with xSPECT Quant™ (Siemens Healthcare, Siemens Erlangen, Germany) which uses the ordered subset conjugate gradient method (OSCGM) [14] to calculate the quantitative activity distribution. The number of iterations (12, 24 or 60) with one subset and 3D-Gaussian post-filter with full width at half maximum (FWHM) of 16.0 mm and 20.8 mm were selected automatically by the reconstruction algorithm, based on the count statistic. Triple energy window scatter correction, CT-based attenuation correction, and resolution recovery were enabled for all image reconstructions. As the xSPECT Quant™ algorithm was not available at the site initially, images of the first patient were quantitatively reconstructed (isotropic voxel size of 3.9 mm^3^) using the OSCGM algorithm with 24 iterations, one subset and a 3D-Gaussian post filter of 10.0 mm FWHM. For these reconstructions, both photo-peak energy windows at 113 keV ± 10% and 208 keV ± 10% were used. All other settings remained as stated above.

### Dosimetry of tumors

The activity in the tumor was determined only in the fourth SPECT image at 72 h p.i., because the signal to noise ratio (SNR) at that scan time was superior compared with the 1, 4 and 24 h p. i. acquisitions. At first a VOI with volume *V*_0_ and activity ${A}_{0}$ enclosing the tumor and its surrounding background was drawn. Subsequently, the threshold was increased until a new VOI with volume $V_{spect}$ and activity $A_{spect}$ separating the tumor activity from background was found. The activity in the tumor at the fourth time point can then be estimated by

$$A_{4} = A_{spect} - \frac{{A_{0} - A_{spect} }}{{V_{0} - V_{spect} }}\left( {V_{spect} - V_{CT} } \right),$$

where the second term is the background correction, with the tumor volume ${V}_{CT}$ determined in the contrast enhanced CT. Spill in and out of activity from the tumor volume into the background was not considered.

Time activity curves (TAC) of the tumors were determined using the mean activity of 3 ml threshold VOIs, drawn in the 1, 4, 24 and 72 h p.i. images. A linear model $y\left(t\right)=\alpha +{\lambda }_{e}\cdot t$ was fitted to the log-transformed data points to estimate the effective half-life ${T}_{e}=ln\left(2\right)/{\lambda }_{e}$. The activity at the first acquisition is then given by

$$A_{1} = A_{4} \cdot e^{{\lambda_{e} \cdot \Delta t}}$$

where $\Delta t$ is the time difference between the first and fourth acquisition. The total number of disintegrations can then be estimated by

$$N = A_{1} \left[ { \frac{{t_{1} }}{2} + \int_{{t_{1} }} {e^{{ - \lambda_{e} t^{\prime } }} dt^{\prime } } } \right]$$

where ${t}_{1}$ is the time of the first acquisition.

The average energy deposition *E* per disintegration in soft tissue voxels of 3.9 mm^3^ and 5.08^3^ mm^3^ with density $\rho = 1\, \frac{{\text{g}}}{{{\text{cm}}^{3} }}$ was calculated with FLUKA, resulting in the mean tumor dose given by $D = \frac{N \cdot E}{{\rho \cdot V_{CT} }}$.

In case the data was not suitable for single exponential fit, N was calculated numerically using trapezoidal integration until the fourth time point and after with analytical integration of the exponential decay using the half-live of Lutetium-177.

### Dosimetry of normal tissues

#### Dosimetry of kidneys

Kidney dosimetry was performed using the dosimetry workflow in the DRT, which includes automatic organ segmentation, time activity curve modelling, and absorbed dose calculation using the medical internal radiation dosimetry (MIRD) method and voxel dosimetry.

After automatic organ segmentation in the 24 h SPECT images, the kidney VOIs were manually corrected to ensure accurate segmentation. Then, the SPECT images at 1, 4 and 72 h where manually co-registered to the 24 h images. Time activity curves were fitted to the measured kidney activity at the four time points using a single exponential function. The calculated and measured data were then used to estimate the absorbed dose in the kidneys using the MIRD method.

#### Dosimetry of stomach

A conservative segmentation approach was adopted for gastric dosimetry to ensure that the VOI included all activity distributed in the gastric wall. TACs were analyzed in the same way as for the tumor dosimetry with a mono-exponential model. The average absorbed dose *D* in the gastric wall was then calculated by using $D=\frac{N\cdot E}{m}$, where *N* is the total number of disintegrations, *E* the average energy deposited in tissue and *m* = 0.15 kg is the mass of the stomach wall.

#### Monte Carlo (MC)-simulation

The energy distribution in ICRP soft tissue of a Lu-177 point source was calculated with the MC-Simulation toolkit FLUKA version 4.1.0. The ICRP soft tissue had a density 1 g cm^−3^. Transport thresholds for electrons and photons were set to 70 keV and 1 keV, respectively. Energy density was scored in a 3.9 mm^3^ and 5.08 mm^3^ binning resembling the voxel size yielding mean energy deposited per disintegration *E* in the center voxel containing the point source 2.34 × 10^–14^ and 2.37 × 10^–14^ J, respectively.

Bone marrow and kidney dosimetry was performed as described before [3].

### PET/CT imaging and reading

SSTR2 targeted PET/CT ([^68^Ga]Ga-DOTATOC or [^68^Ga]Ga-DOTATATE), acquired before study participation, was available for all patients. PET/CT imaging was performed according to the EANM guideline [15].

Visual evaluation of SPECT/CT and PET/CT imaging as well as SUV measurements were performed using Syngo Via software (Syngo Via version VB40B, Siemens Healthcare, Erlangen, Germany) by a board certified nuclear medicine physician (CR) who had > 15 years experience. The reader had access to all available patient and imaging data to enable direct comparison of lesions. SUVmean liver for the SSTR2 PET/CT ratio was measured in the right liver lobe in a 15 ml volume of interest.

### Statistics

Sample size was estimated to be able to show a difference in mean tumor uptake with and without previous intake of Sacubitril with at least 90% power at a significance level of 5%. Assuming independent tumor uptake of [^177^Lu]Lu-PP-F11N in the same patient and a tumor detection rate of 0–3 tumours per patient, a total of 8 patients should be recruited. The difference of stability in blood as well as tumor and organ-absorbed dose of [^177^Lu]Lu-PP-F11N with and without Entresto^®^ was tested using a matched paired Wilcox test (Wilcoxon’s signed rank test).

## Results

Demographics of patients are summarized in Table 1.

**Table 1:** Patient characteristics

	Patient 1	Patient 2	Patient 3	Patient 4
Gender	Male	Male	Female	Female
Initial tumor stage	pT4a pN1b M1 V1 Pn0 R1	pT4a N1b L1 V1 Pn0	pT4a N1a L0 V0	pT3 N1b M0
Molecular pathology/RET	Sporadic, RET mutation p.M918T	Sporadic, RET mutation p.M918T	Unknown	Sporadic, RET negative
Age at diagnosis	40 years	49 years	47 years	22 years
Time since diagnosis	13.8 months	4.5 months	113.4 months	143.4 months
Previous therapy	Thyroidectomy and neck dissection Vandetanib 300 mg/d	Thymus resection, thyroidectomy, and neck dissection radiation therapy	Thyroidectomy and neck dissection Atypical liver resection (metastases)	Thyroidectomy and neck dissection Vandetanib. ^177^Lu-DOTATOC (30 GBq). Radiation therapy neck
Time since last tumor therapy	0.5 months	4.5 months	11.2 months	8.5 months
Calcitonin	114 pmol/l	349 pmol/l	153 pmol/l	14,000 pmol/l
CEA	8.7 µg/l	107 µg/l	13.4 µg/l	60.7 µg/l

	Patient 5	Patient 6	Patient 7	Patient 8
Gender	Male	Male	Male	Male
Initial tumor stage	pT3a N0 M1	pT2 N1b M0 L1 V1 R0	pT1b N1b cM0 L1 V1 Ro	T1a (m) N1 M0
Molecular pathology/RET	Sporadic, RET substitution p.G691S	MEN IIA, RET mutation p.C611Y	Germline negative, somatic unknown	MEN2, RET mutation p.S891A
Age at diagnosis	48 years	40 years	20 years	35 years
Time since diagnosis	14.6 months	41.8 months	138.6 months	178 months
Previous therapy	Thyroidectomy and neck dissection Radiation therapy zoledronate	Thyroidectomy and neck dissection	Thyroidectomy and neck dissection PRRT (^177^Lu-DOTATATE, 30 GBq) radiation therapy	Thyroidectomy and neck dissection
Time since last tumor therapy	5.4 months	41.8 months	13.2 months	178 months
Calcitonin	46.8 pmol/l	441 pmol/l	2840 pmol/l	30 pmol/l
CEA	22.1 µg/l	48.7 µg/l	117 µg/l	4 µg/l

Patient characteristics of all patients at the time of study inclusion

### Acute toxicity

Infusion of [^177^Lu]Lu-PP-F11N was tolerated well in all patients with acute, self-limiting toxicity of maximal grade 2 according to CTCAE and comparable to the effects as described before [3]. Adverse effects with and without Entresto^®^ premedication did not significantly differ (Table 2). No serious adverse event or suspected unexpected serious adverse reaction occurred.

**Table 2:** Adverse events

Patient no	Definition	Grade w/o	Grade w	Patient no	Definition	Grade w/o	Grade w
1	Hot flashes	1	1	6	Hypocalcemia	1	–
1	Flush	1	1	6	Hypokalemia	1	–
1	Nausea	1	1	6	Headache	1	–
1	Dizziness	1	1	6	Hot flashes	1	–
1	Sweating	1	–	6	Nausea	1	1
1	Hypocalcemia	–	2	6	Flushing	1	1
2	Hypocalcemia	1	–	6	Hyperhidrosis	–	1
2	Dizziness	1	1	7	Flushing	1	1
2	Hyperhidrosis	1	1	7	Nausea	1	1
3	Abdominal pain	1	–	7	Vomiting	1	–
3	Paresthesia	1	–	7	Hyperhidrosis	–	1
3	Hot flashes	–	1	7	Hypokalemia	–	1
3	Dizziness	1	1	7	Fatigue	–	1
3	Nausea	1	1	8	Nausea	1	1
4	Hot flashes	1	1	8	Headache	1	1
4	Neck pain	1	1	8	Dizziness	–	1
5	Paresthesia	–	1				

Adverse effects without (w/o) and with (w) Entresto^®^ premedication, graded according to CTCAE version 4.03

### In-vivo stability

**Fig. 1:**
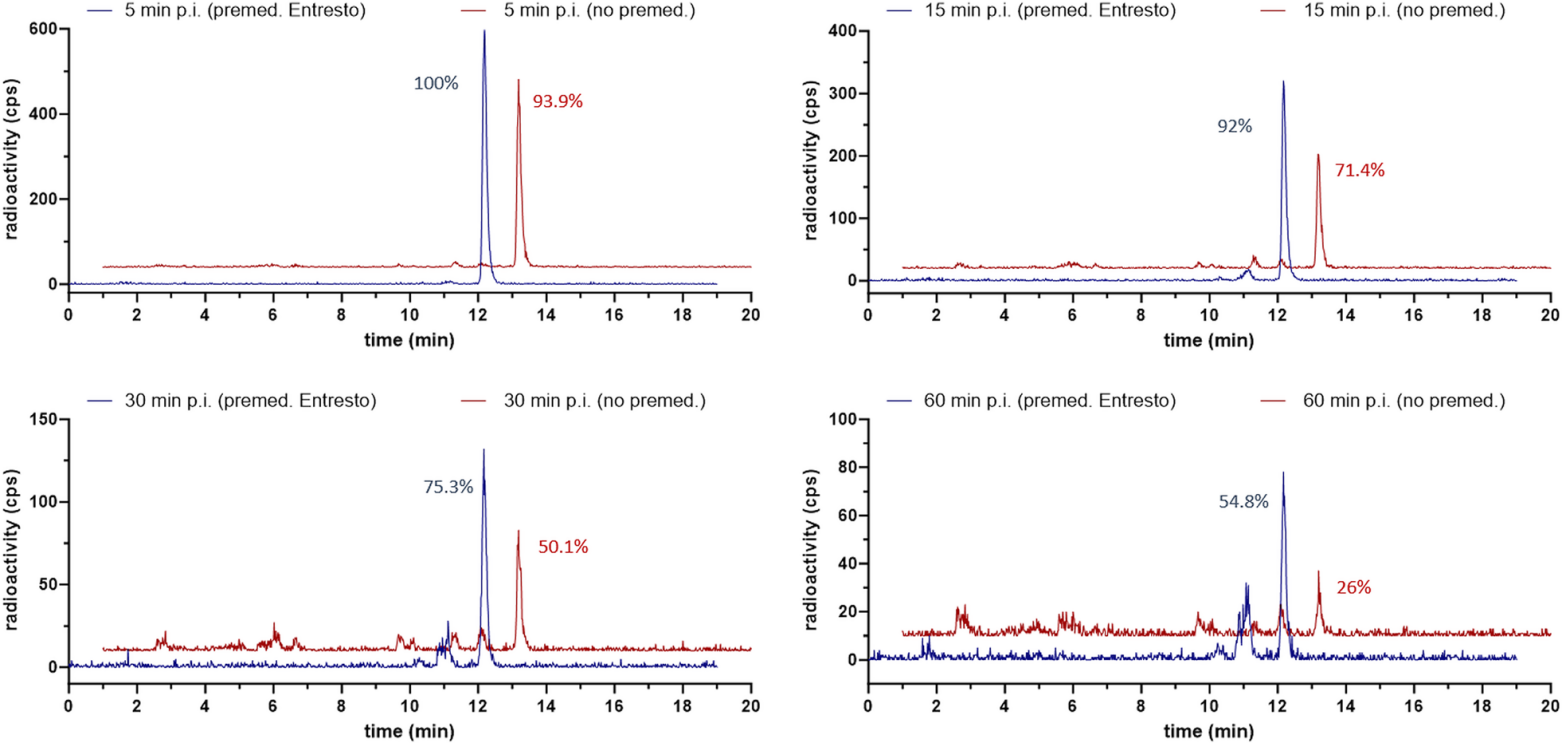
In-vivo stability of of [^177^Lu]Lu-PP-F11N. Representative HPLC chromatograms obtained from blood stability studies of patient 7. These chromatograms serve as illustrative examples. A direct comparison of the chromatograms at each time point reveals a notably higher percentage of intact peptide following premedication with Entresto^®^. Blue line: with Entresto^®^, red line: without Entresto^®^

In-vivo stability of [^177^Lu]Lu-PP-F11N was significantly higher (*p* < 0.001) after Entresto^®^ premedication. Median stability at 5, 15, 30 and 60 min p.i. was 1.1, 1.1, 1.2 and 1.6-fold higher, compared to the stability without premedication. No distinct metabolites were identifiable. For details, see Fig. 1 and Table 3.

**Table 3:** In-vivo stability of [^177^Lu]Lu-PP-F11N

Time p.i. (min)	Entresto^®^	Stability (% of initial signal) in patients 1–8									
		1	2	3	4	5	6	7	8	Median	IQR
5	w/o	71	95.5	n.a.^a^	96.1	94.3	96.5	93.9	92.4	94.1	2.7
	w	91.4	99.4	98.3	n.a.^b^	99.6	99.9	100	98.3	99.5	1.5
15	w/o	69.9	80.3	91.4	90.5	88.8	90.2	71.4	68.1	80.3	19.3
	w	92.2	96.7	92.1	n.a.^b^	91.6	99.2	92	88.9	92.1	2.7
30	w/o	64.5	72.3	74.8	67.6	n.a.^a^	60.3	50.1	n.a.^a^	64.5	9.8
	w	78.5	85.7	80.6	n.a.^b^	85.5	77.9	75.3	79.9	78.5	4.9
60	w/o	37.9	47.1	44.3	34.3	n.a.^a^	53.7	26	n.a.^a^	41.1	11.2
	w	63.5	70.3	64.8	n.a.^b^	75.6	n.a.^b^	54.8	58.8	64.15	10

In-vivo stability of [^177^Lu]Lu-PP-F11N without (w/o) and with (w) Entresto^®^ premedication at 5, 15, 30 and 60 min p.i. Values with premedication are significantly higher (*p* < 0.001)

^a^Activity rate too low for HPLC analysis

^b^HPLC malfunction

### Whole body scintigraphy and SPECT/CT imaging

**Fig. 2:**
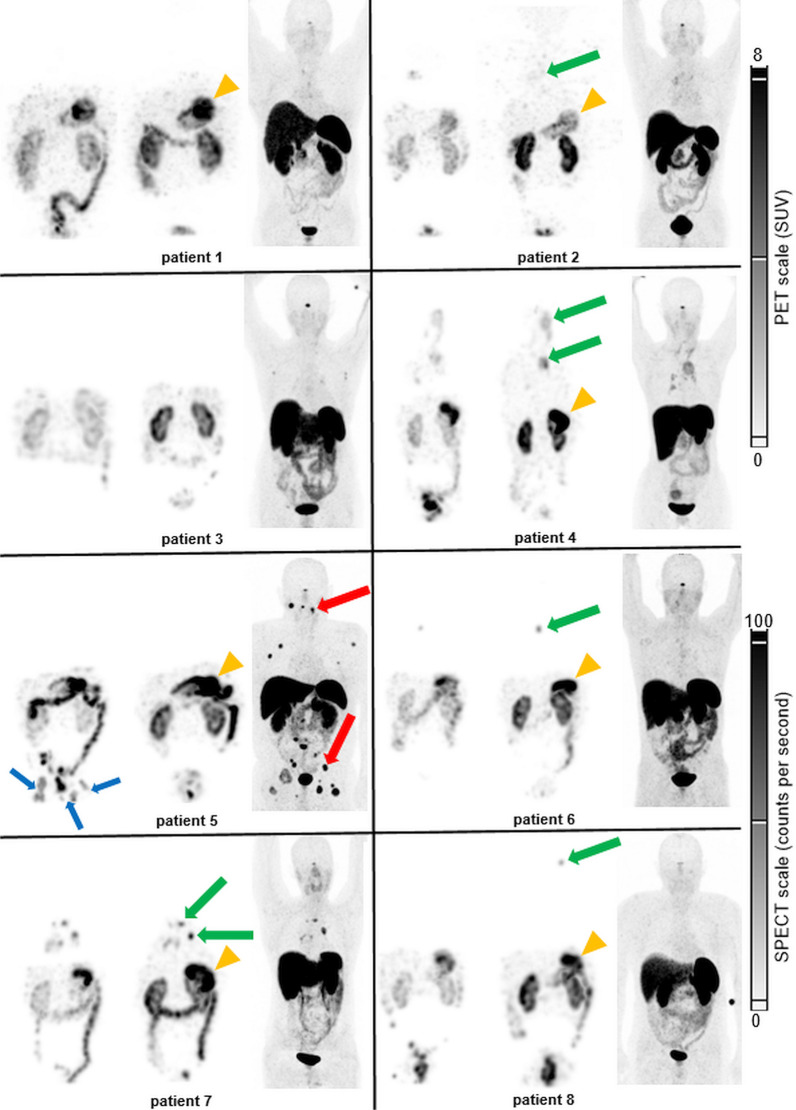
[^177^Lu]Lu-PP-F11N and SSTR2 imaging in all patients. [^177^Lu]Lu-PP-F11N SPECT maximum intensity projection (MIP) of patient 1–8 at 24 h p.i. without (left) and with (middle) Entresto^®^ premedication. MIP of the corresponding SST2R PET/CT for each patient (right). Green arrows: MTC metastases, visible in [^177^Lu]Lu-PP-F11N scintigraphy. Red arrows: MTC metastases only visible in SST2R PET/CT in a single patient (only two of multiple metastases labelled). Blue arrows: urine contamination. Orange arrowheads: physiological stomach uptake. Furthermore, uptake in kidneys, bowels and urinary bladder is visible in all patients

In five of eight consecutive patients, tumor uptake of [^177^Lu]Lu-PP-F11N was visible (Fig. 2 and Table 4). [^68^Ga]Ga-DOTATOC or [^68^Ga]Ga-DOTATATE PET/CT, performed within 12 weeks before study inclusion, was positive in four of eight patients.

**Table 4:** Comparison CKK2R and SSTR targeted uptake

Patient	Visible tumor uptake		CCK2R dosimetry (Gy/GBq)		SSTR2R PET/CT		Indication for PRRT	
	CCK2R	SST2R	w/o	w	SUVmax	SUVratio	CCK2R^c^	SST2R
1	No	No	n.a	n.a	n.a	n.a	No	No
2	Yes	Yes	n.a	n.a	3.7	0.5	No	No
3	No	No	n.a	n.a	n.a	n.a	No	No
4	Yes	Yes	0.03	0.05	2.3	0.25	No	No
4^a^	Yes	Yes	0.05	0.07	4.8	0.5		
5	No	Yes	n.a	n.a	19.8^b^	1.8^b^	No	Yes
6	Yes	No	0.99	1.26	n.a	n.a	Yes	No
7	Yes	Yes	0.75	1.12	12.9	1.6	Yes	Yes
7^a^	Yes	Yes	0.22	0.47	10.9	1.3		
8	Yes	No	0.33	0.73	n.a	n.a	Yes	No

Visible tracer uptake in tumor in CCK2R scintigraphy and SPECT/CT and in SSTR targeted PET/CT. Tumor doses of [^177^Lu]Lu-PP-F11N without (w/o) and with (w) Entresto^®^ and SUVmax, respectively ratios of SUVmax lesion to SUVmean liver of SST2R PET/CT

^a^2^nd^ tumor in the same patient

^b^Measured in “hottest” tumor lesion

^c^Potential indication for CCK2R PRRT based on visual interpretation, since there are currently no standards for the indication of CCK2R PRRT

**Fig. 3:**
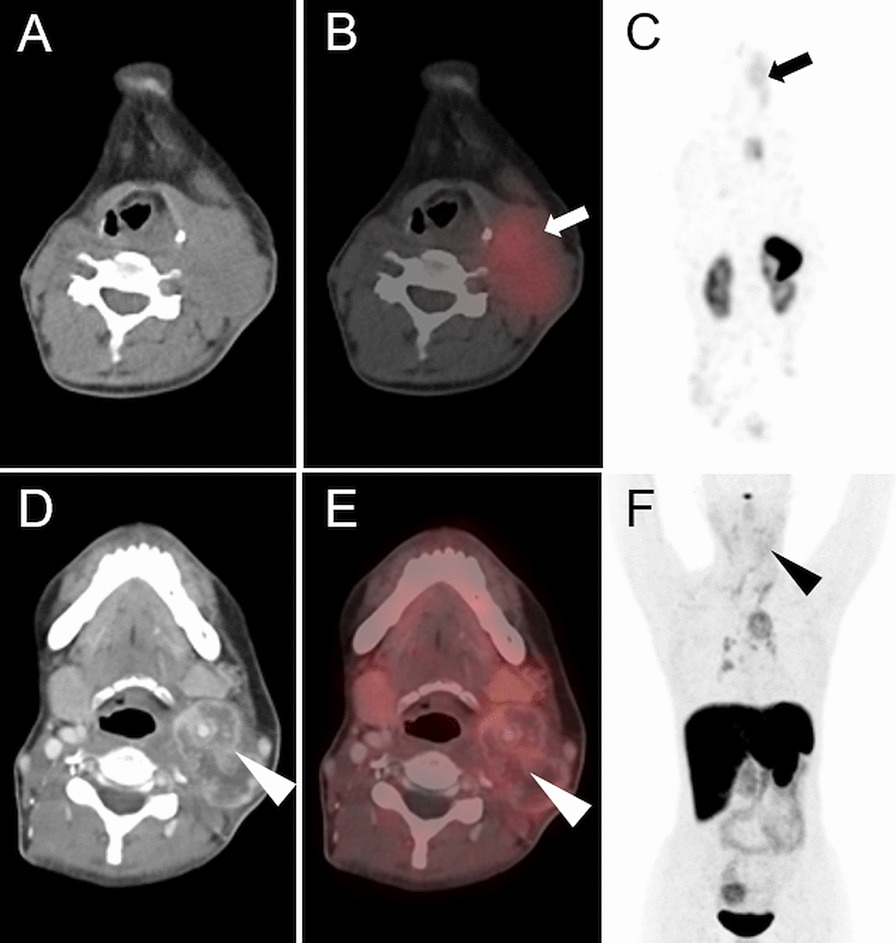
Effect of premedication on the [^177^Lu]Lu-PP-F11N uptake in patient 4. Axial CT, SPECT/CT and MIP of [^177^Lu]Lu-PP-F11N SPECT/CT (**A**–**C**) and [^68^Ga]Ga-DOTATOC PET/CT (**D**–**F**) of patient 4. Uptake of [^177^Lu]Lu-PP-F11N (arrows, with Entresto^®^ premedication) and [^68^Ga]Ga-DOTATOC (arrowheads) in the predominantly necrotic lymph node metastasis with only low intensity

In patient 2, the combination of low tumor uptake of [^177^Lu]Lu-PP-F11N and overlay with adjacent vessels hindered tumor dosimetry. In the remaining four patients with visible tumor uptake, tumor dosimetry was performed in up to two tumor lesions per patient (n = 6). The lowest tumor absorbed dose was measured in patient 4 with large, predominantly necrotic lymph node metastases and low uptake also seen in [^68^Ga]Ga-DOTATOC PET/CT (Figs. 2 and 3).

**Fig. 4:**
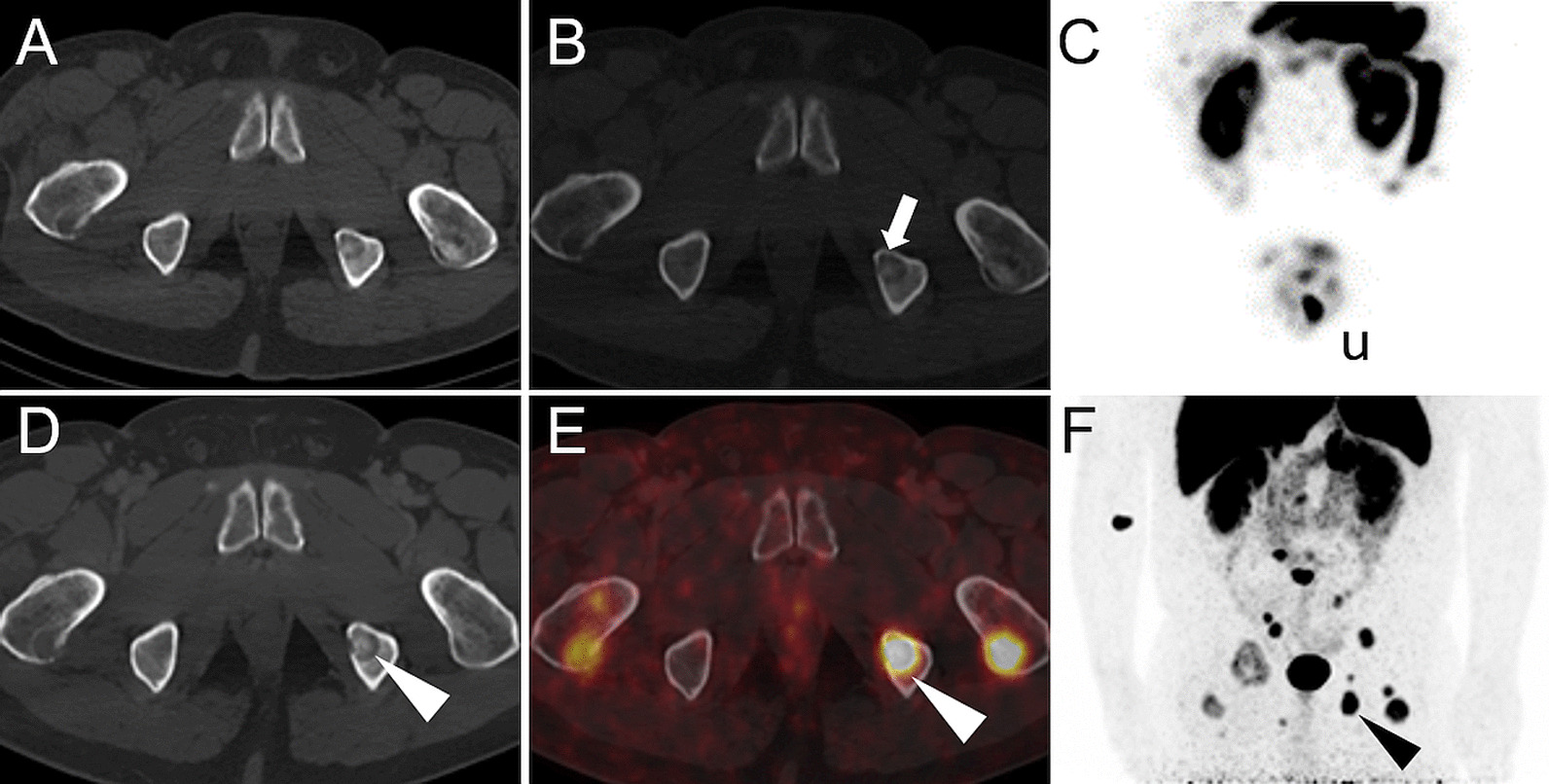
Discrepancy of [^177^Lu]Lu-PP-F11N and [^68^Ga]Ga-DOTATOC uptake in patient 5. Axial CT (**A**, **D**), SPECT/CT (**B**), PET/CT (**E**) and MIP (**C**, **F**) of patient 5. No visible uptake of [^177^Lu]Lu-PP-F11N in SPECT/CT (white arrow, **B**, with Entresto^®^ premedication) and MIP (**C**). Intense uptake of [^68^Ga]Ga-DOTATOC in multiple lytic bone metastases (white and black arrowheads, **E**, **F**. u: urine contamination

**Fig. 5:**
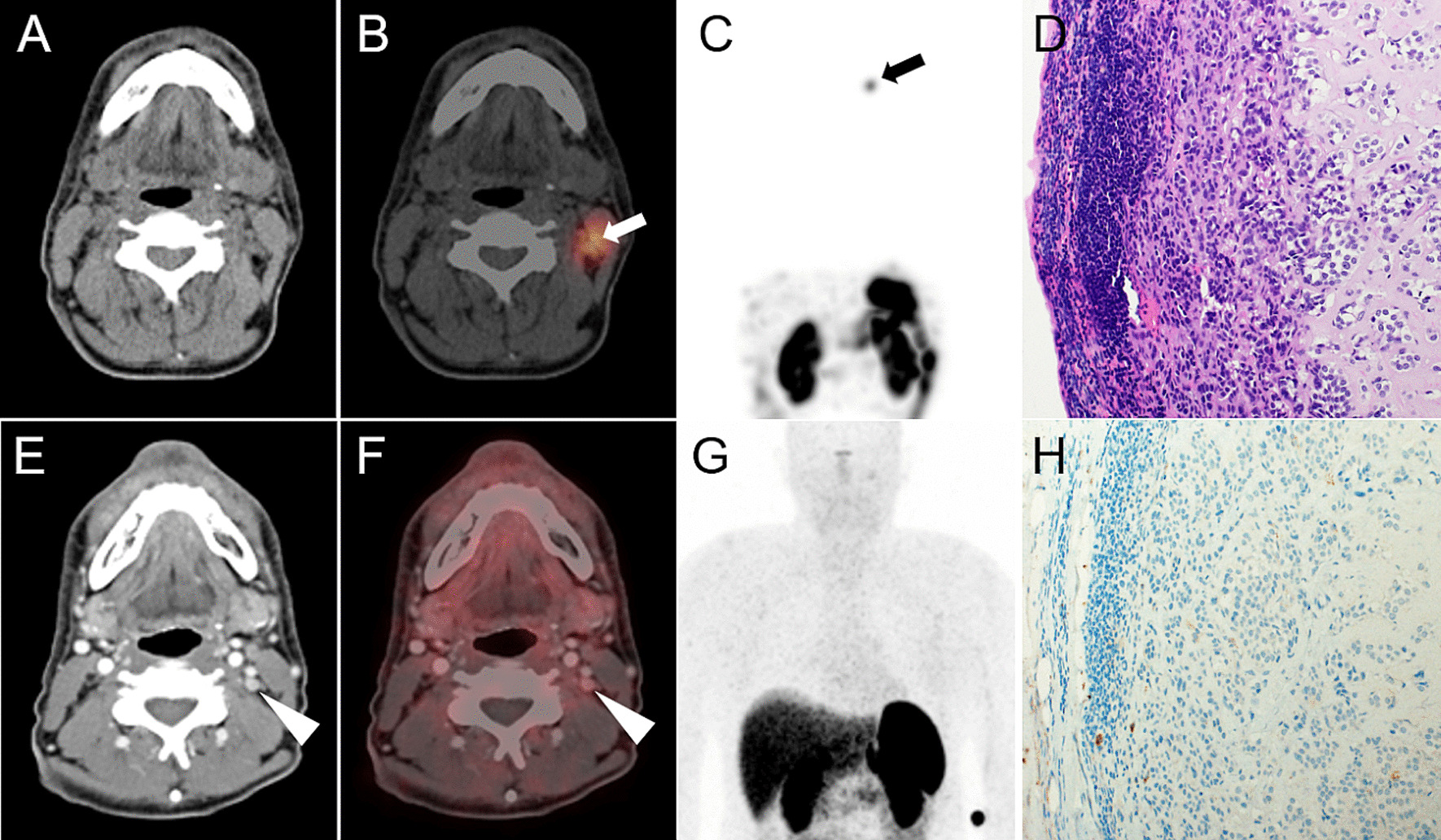
Detection of a lymph node metastasis by [^177^Lu]Lu-PP-F11N SPECT/CT in patient 8. Corresponding axial CT (**A**), [^177^Lu]Lu-PP-F11N SPECT/CT (**B**) and MIP (**C**) of patient 8, showing focal tracer uptake in a left cervical lymph node (arrows) only after Entresto^®^ pretreatment (see Fig. 2). Corresponding images (**E**–**G**) of the ^68^Ga-DOTATOC PET/CT without visible tracer uptake (arrowheads). The corresponding lymph node is visible in the contrast medium enhanced CT (**E**, 6 mm). Hematoxylin and Eosin (**D**) and SSTR2 (**H**) staining of the lymph node after resection, showing metastasis of MTC, negative for SSTR2

In two patients (patient 1 and 3) with a calcitonin level of 114 and 153 pmol/l both, [^177^Lu]Lu-PP-F11N SPECT/CT and [^68^Ga]Ga-DOTATOC PET/CT were negative (Fig. 2). In patient 5, tracer uptake of histologically confirmed metastases was only present with [^68^Ga]Ga-DOTATOC PET/CT, but not with [^177^Lu]Lu-PP-F11N SPECT/CT (Figs. 2 and 4). In two patients (patient 6 and 8), tumor uptake was only present for [^177^Lu]Lu-PP-F11N, but not in [^68^Ga]Ga-DOTATOC PET/CT (Figs. 2 and 5). Patient 8 underwent surgery and histology confirmed a SSTR2-negative lymph node metastasis of MTC (Fig. 5). For results and comparison of SSTR2 PET/CT to CCK2R targeted imaging, see Table 4.

Available CT imaging did not reveal any lesions suspicious for metastases that were not visible in CCK2R and/or SSTR2-targeted imaging. On the other hand, CCK2R imaging revealed lesions suspect for MTC metastasis, but not suspected in CT imaging, in patient 6 and patient 8.

### Tumor and organ absorbed doses

Tumor dosimetry was possible in four patients. In the remaining patients, absence of pathological tracer uptake in [^177^Lu]Lu-PP-F11N SPECT/CT imaging (n = 3) or the combination of low tumor uptake with insufficient discrimination to blood pool activity (n = 1) prevented dosimetry (Table 5). In all patients with tumor dosimetry, tumor absorbed doses after Entresto^®^ premedication were higher (median: 2.6-fold), compared to tumor absorbed doses without premedication.

**Table 5:** Radiation doses

Patient	Mean tumor dose (Gy/GBq)		Kidney dose (Gy/GBq)		Stomach dose (Gy/GBq)		Bone marrow dose (Gy/GBq)	
Entresto^®^	Without	With	Without	With	Without	With	Without	With
1	n.a	n.a	0.04	0.07	0.3	0.48	0.02	0.02
2	n.a	n.a	0.05	0.13	0.26	0.43	0.04	0.04
3	n.a	n.a	0.05	0.12	0.03	0.05	0.03	0.04
4	0.03	0.05	0.05	0.12	0.23	0.50	0.03	0.05
4^a^	0.05	0.07	0.05	0.12	0.23	0.50	0.03	0.05
5	n.a	n.a	0.03	0.07	1.15	1.53	0.02	0.03
6	0.99	1.26	0.05	0.11	0.19	0.30	0.04	0.06
7	0.22	0.75	0.04	0.10	0.17	0.88	0.03	0.04
7^a^	0.47	1.12	0.04	0.10	0.17	0.88	0.03	0.04
8	0.33	0.73	0.03	0.09	0.2	0.39	0.03	0.04
Median	0.28	0.74	0.05	0.11	0.2	0.5	0.03	0.05
IQR	0.34	0.79	0.01	0.04	0.05	0.37	0.002	0.014
Wilcoxon’s signed rank test	0.03	0.01	0.01	0.01	0.22 NS	0.19 NS	0.44 NS	

Patient	Tumor-to-kidney dose ratio		Tumor-to-stomach dose ratio		Tumor-to-bone marrow dose ratio	
Entresto^®^	Without	With	Without	With	Without	With
1	n.a	n.a	n.a	n.a	n.a	n.a
2	n.a	n.a	n.a	n.a	n.a	n.a
3	n.a	n.a	n.a	n.a	n.a	n.a
4	0.6	0.4	0.1	0.1	1.1	1
4^a^	1.0	0.6	0.2	0.1	1.8	1.4
5	n.a	n.a	n.a	n.a	n.a	n.a
6	19.8	11.5	5.2	4.2	24.8	21.4
7	5.5	7.5	1.3	0.9	7.3	20.3
7^a^	11.8	11.2	2.8	1.3	15.7	30.3
8	11.0	8.1	1.7	1.9	11	17.8
Median	8.3	7.8	1.5	1.1	9.2	19.1
IQR	9.5	8.1	2.1	1.5	11.4	15.7
Wilcoxon’s signed rank test						

Radiation doses of tumors and organs and tumor-to-organ dose ratios without and with Entresto^®^ premedication. *n.a.* not applicable (no measurable tumor), *NS* not significant

^a^2nd tumor in the same patient

Dosimetry of stomach, kidneys and bone marrow was possible in all eight patients. Median absorbed doses of these organs after premedication were 2.1, 2.4 and 1.4–fold higher, compared to the absorbed doses without premedication, but without an increase in adverse events. Tumor-to-organ absorbed dose ratios did not change significantly after premedication. For the complete results of dosimetry see Table 5.

## Discussion

The main results of this study indicate a relevant stabilization of [^177^Lu]-PP-F11N in patients by pretreatment with Entresto^®^, resulting in higher radiation doses in tumors, but also in other organs and without revealing additional toxicity. These findings support the hypothesis that [^177^Lu]Lu-PP-F11N shows only moderate stability in humans due to premature cleavage by NEP.

One option to improve stability and therefore efficacy is the further radiochemical modification of the structure of [^177^Lu]Lu-PP-F11N. Alternatively, the premedication with the approved drug Entresto^®^, containing the NEP-inhibitor sacubitril, might be a different, more straightforward approach. Our data show an up to 2.6-fold increased absorbed tumor dose after premedication. Furthermore, in patient 8 the identification of a lymph node metastasis, enabling for tumor surgery, was only possible after premedication (Fig. 5). Consequently, premedication could enable for the administration of a lower activity of [^177^Lu]Lu-PP-F11N for PRRT, saving [^177^Lu]LuCl3, reducing radiation burden to patients and staff, as well as radioactive waste. It is likely that this would be a highly cost-effective procedure since Entresto^®^ costs less than 3 Euro/dose.

Acute toxicity of [^177^Lu]Lu-PP-F11N with or without Entresto^®^ premedication did not differ. Also delayed, radiation induced organ toxicity after premedication can be expected to be equal to the alternative use of a corresponding, higher activity of [^177^Lu]Lu-PP-F11N for PRRT. A disadvantage of the use of Entresto^®^ for premedication is the fact, that this medication not only contains the NEP inhibitor sacubitril, but also the angiotensin-receptor blocker valsartan. This was mainly due to regulatory issues, since Entresto is approved in Switzerland whereas other NEP-inhibitors such as racecadotril are not. A recent clinical study suggests that premedication with 400 mg racecadotril 90 min before infusion of [^111^In]In-DOTA-minigastrin11 resulted in 2.5 to 4.4-fold higher uptake in MTC tumor lesions and might therefore be an interesting alternative to Entresto^®^ [16]. Importantly for our approach, the additional anti-hypertensive compound valsartan did not significantly increase adverse events in our pilot study. Our results contrast with the published lack of effect of phosphoramidon co-administration on [^177^Lu]Lu-PP-F11N uptake in CCK2R positive tumors in mice [7]. This indicates that the predictive value of preclinical stability testing of radiolabeled peptides might be limited, e.g. on account of different peptidase expression between species. In the given situation of a moderately stable compound with high in-vitro stability, this could be of even greater relevance as it may only become apparent in a situation of a “maximum stress load”, e.g. due to the abundant and ubiquitous presence of NEP in the human body under physiological conditions.

Regarding the absorbed kidney doses, tubular reabsorption of [^177^Lu]Lu-PP-F11N by the megalin receptor must be suspected [17]. In this situation, NEP inhibition within the renal brush border membrane might result in increased tubular resorption [18]. This is an effect that should be considered in general in the context of peptidase inhibition for PRRT. However, the increase of absorbed kidney doses after premedication in our study was within the same range as the absorbed doses in tumor and stomach and below dose limiting values. Therefore, we do not consider such an effect as limiting in the situation of NEP inhibition for PRRT with [^177^Lu]Lu-PP-F11N.

In contrast to our previously published data [3], the current study did not detect CCK2R positive tumors in 3 patients with MTC. As SSTR2 PET/CT failed to detect tumor lesions in two of these 3 patients, negativity in CCK2R imaging in these patients may be attributed to small tumor volumes. An interesting finding are the intensively positive bone metastases in [^68^Ga]Ga-DOTATOC PET/CT in the third CCK2R imaging negative patient. Molecular investigation of this patient revealed a somatic mutation in the RET gene (substitution p.G691S). Further basic research should be performed to define whether this mutation contributed to the different receptor expression in patients. In this small pilot study, we were not able to identify predictive factors for CCK2R positivity and there was no previous patient selection, e.g. according to the CCK2R expression status. Therefore, only 3 of 8 patients showed tumor absorbed doses which were suitable for [^177^Lu]Lu-PP-F11N therapy in combination with Entresto^®^. At the same time only 2 patients, including one CCK2R negative patient, are candidates for SSTR2 directed PRRT. Therefore, CCK2R and/or SSTR2 imaging might be critical for the selection of the most suitable target for PRRT. The identification not only of RET mutations in tumor tissue, but also of predictive indicators for the efficacy of PRRT, for example CCK2R and SSTR2 imaging, will likely be of great importance for the choice of the most appropriate therapy of MTC on an individual level.

The dose and timing of premedication with Entresto^®^ in this study was chosen in accordance with the available pharmacokinetic data and the recommended dosing in patients [13]. This approach resulted in a significant NEP inhibition without additional adverse events. However, even greater effects may be achieved by optimizing the dose and timing of premedication. Further evaluation of the optimal scheduled peptidase inhibitor premedication for PRRT should be considered in future studies in case of a broader clinical implementation of this approach.

A limitation of our study is the small number of patients with tumor lesions appropriate for dosimetry and in consequence a small sample size for statistical comparison of the tumor dose with and without Entresto^®^ premedication. However, the patient numbers were within the limits of the previously performed sample size calculation. Furthermore, the statistical comparison of the stomach-absorbed dose confirmed significant higher doses after premedication. Stomach-absorbed dose can be considered as specific control measurement for specific uptake because of the high expression of CCK2R on neuroendocrine cells in the gastric mucosa [19].

## Conclusions

Herein, we present the results of the to our knowledge first clinical study demonstrating the feasibility and safety of stabilization of a radiopharmaceutical already in clinical evaluation for PRRT (clinicaltrials.gov: NCT02088645) via premedication with a protease inhibitor. Premedication with Entresto^®^ is safe for patients receiving [^177^Lu]Lu-PP-F11N and results in stabilization of the radiopharmaceutical via inhibition of NEP. Consecutively, higher absorbed tumor doses, but not higher tumor-to-organ dose ratios can be achieved. Nevertheless, this approach can reduce the activity of [^177^Lu]Lu-PP-F11N that must be administered for PRRT.

This study also shows the heterogenous biology of MTC as evidenced by the different expression of receptors (CCK2R and SST2R). The molecular background of this finding remains to be established, similarly the possible prognostic value. This study, however, indicates that the appropriate receptor imaging—i.e. CCK2R or SST2R imaging—should be performed prior to targeted therapy.

## Data Availability

The datasets used and/or analysed during the current study are available from the corresponding author on reasonable request.
